# An Investigation of the Altered Textural Property in Woody Breast Myopathy Using an Integrative Omics Approach

**DOI:** 10.3389/fphys.2022.860868

**Published:** 2022-03-17

**Authors:** Amelia A. Welter, Wan Jun Wu, Ryan Maurer, Travis G. O’Quinn, Michael D. Chao, Daniel L. Boyle, Erika R. Geisbrecht, Steve D. Hartson, Brian C. Bowker, Hong Zhuang

**Affiliations:** ^1^Department of Animal Sciences and Industry, Kansas State University, Manhattan, KS, United States; ^2^Division of Biology, Kansas State University Microscopy Facility, Manhattan, KS, United States; ^3^Department of Biochemistry and Molecular Biophysics, Kansas State University, Manhattan, KS, United States; ^4^Department of Biochemistry and Molecular Biology, Oklahoma State University, Stillwater, OK, United States; ^5^United States National Poultry Research Center USDA, Agricultural Research Service, Athens, GA, United States

**Keywords:** calcium, lipidomics, proteomics, sarcoplasmic reticulum, woody breast, proteolysis

## Abstract

Woody breast (WB) is a myopathy observed in broiler *Pectoralis major* (PM) characterized by its tough and rubbery texture with greater level of calcium content. The objective of this study was to investigate the functionality/integrity of WB sarcoplasmic reticulum (SR), which may contribute to the elevated calcium content observed in WB and other factors that may influence WB texture. Fourteen Ross line broiler PM [7 severe WB and 7 normal (N)] were selected, packaged, and frozen at −20°C at 8 h postmortem from a commercial processing plant. Samples were used to measure pH, sarcomere length, proteolysis, calpain activity, collagenase activity, collagen content, collagen crosslinks density, and connective tissue peak transitional temperature. Exudate was also collected from each sample to evaluate free calcium concentration. The SR fraction of the samples was separated and utilized for proteomic and lipidomic analysis. The WB PM had a higher pH, shorter sarcomeres, lower % of intact troponin-T, more autolyzed μ/m calpain, more activated collagenase, greater collagen content, greater mature collagen crosslinks density, and higher connective tissue peak transitional temperature than the N PM (*p ≤* 0.05). Exudate from WB PM had higher levels of free calcium than those from N PM (*p <* 0.05). Proteomics data revealed an upregulation of calcium transport proteins and a downregulation of proteins responsible for calcium release (*p <* 0.05) in WB SR. Interestingly, there was an upregulation of phospholipase A2 (PLA2), and cholinesterase exhibited a 7.6-fold increase in WB SR (*p <* 0.01). Lipidomics data revealed WB SR had less relative % of phosphatidylcholine (PC) and more lysophosphatidylcholine (LPC; *p <* 0.05). The results indicated that upregulation of calcium transport proteins and downregulation of calcium-release proteins in WB SR may be the muscle’s attempt to regulate this proposed excessive signaling of calcium release due to multiple factors, such as upregulation of PLA2 resulting in PC hydrolysis and presence of cholinesterase inhibitors in the system prolonging action potential. In addition, the textural abnormality of WB may be the combined effects of shorter sarcomere length and more collagen with greater crosslink density being deposited in the broiler PM.

## Introduction

To keep up with the high demand for broiler breast meat, the poultry industry has been selecting birds for rapid growth rate and increases in overall body weight, muscle yield, and feed conversion efficiency (Petracci et al., 2015). However, the improvement in production efficiency has also led to the emergence of a number of meat quality issues (Yalcin et al., 2018), most notably the Woody Breast (WB) syndrome (Tijare et al., 2016). This abnormality is typically identified by its tough, rubbery texture in the cranial region and a hard ridge-like bulge along the caudal region of the *Pectoralis Major* (PM) muscle (Sihvo et al., 2014; Kuttappan et al., 2016). Furthermore, many studies have consistently shown elevated levels of free sarcoplasmic calcium (Soglia et al., 2016; Tasoniero et al., 2016) and increased pH (Mudalal et al., 2015; Cai et al., 2018; Baldi et al., 2020) in WB PM. Elevated levels of calcium in WB broilers could result in an overactive calcium-dependent protease system (Ohlendieck, 2013), causing oxidative stress and metabolic disorders associated with the development of hypoxic conditions in the PM (Mutryn et al., 2015; Abasht et al., 2016; Dridi and Kidd, 2016). Consequently, these conditions may further lead to multifocal regenerative myodegeneration and necrosis resulting the increased scar tissues deposition in PM (Sihvo et al., 2014). In addition, proteolytic enzyme activity during rigor mortis and postmortem storage of meat is calcium-, temperature-, and pH-dependent (Maddock et al., 2005; Koohmaraie and Geesink, 2006). However, the effect of increased free sarcoplasmic calcium and pH on muscle contraction and proteolytic enzyme activity as well as muscle necrosis on collagen characteristics are not well understood in WB PM.

The sarcoplasmic reticulum (SR) is an organelle that is responsible for the uptake, storage, and release of calcium within muscle cells. Various proteins are involved in the resequester, release, and storage of calcium from the SR, with the main ones being sarco(endo)plasmic reticulum Ca^2+^-ATPase (SERCA), calsequestrin (CASQ), and ryanodine receptors (RyR), respectively (Barone et al., 2015). In addition to the proteins, loss in phospholipid bilayer membrane integrity could also be a source of calcium leakage and affecting the functionality of the proteins anchored to the membrane (Rossi et al., 2008). Phospholipase A2 (PLA2) is a ubiquitous enzyme that is activated by calcium resulting in phospholipid cleavage into lysophospholipids and free fatty acids (Burke and Dennis, 2009). It is possible that one or more proteins/phospholipids are accountable for the alteration of SR functionality, resulting in the accumulation of sarcoplasmic free calcium. Therefore, the objective of this study was to investigate the functionality/integrity of WB SR and other factors contributing to the elevated calcium content observed in WB as well as the enzymatic activities that may influence WB texture during rigor mortis and postmortem aging.

## Materials and Methods

### Sample Collection, Fabrication, and Preparation

**Figure 1:**
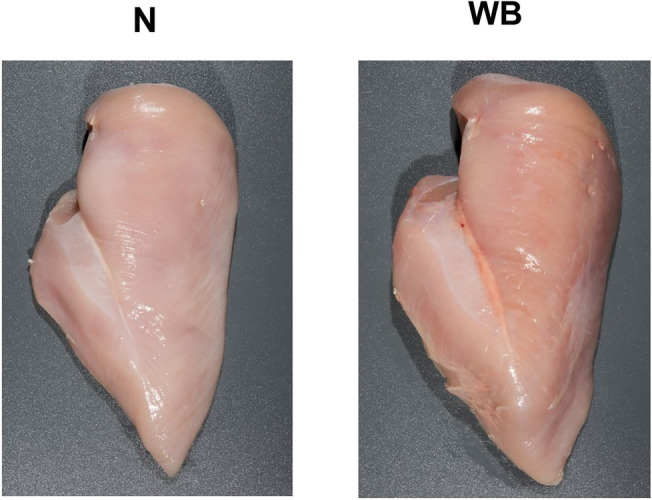
Representative images of Woody Breast (WB) and normal (N) Pectoralis Major muscle utilized in this study.

Skinless, boneless broiler (male, Ross 708, slaughter age 54–58 days, liveweight 4.1–4.3 kg) PM samples were collected from a commercial processing facility that utilized electrical stunning to render broilers unconscious. All PM samples were collected postchill from the deboning line at approximately 3 h postmortem. The PM samples were weighed and scored for woody breast at approximately 6 h postmortem. Each sample was assigned a normal, moderate, or severe score for WB based on the degree of observable hardness. Seven PM samples that exhibited severe WB and seven PM samples exhibiting zero signs of WB (N) were selected for analysis (Figure 1). At 8 h postmortem, samples were vacuum packaged and stored at −20°C until shipment to Kansas State University Meat and Muscle Biology Laboratory. All PM samples were thawed in a refrigerator at 4°C for 24 h. The exudate (~10 ml) was collected from each sample for calcium content analysis. Two 1.9 cm × 1.9 cm strips were removed from the cranial end of the PM samples. The slices included the entire depth of the PM. The first 1.9 cm strip was frozen in liquid nitrogen and pulverized using a blender (model 51BL32; Waring Commercial, Stamford, CT) for lab analysis, and the second strip was repackaged for SR extraction and stored at −80°C until analysis.

### pH Determination

Five gram of pulverized PM and 50 ml of ultrapure water were homogenized at 10,000 rpm for 20 s using a bench top homogenizer in duplicate. The pH value was determined using a pH probe (InLab Science Pro-ISM; Mettler Toledo, Columbus, OH) attached to a pH meter (SevenCompact pH; Mettler Toledo) while solution was stirred constantly using a magnetic stir bar. The pH probe was calibrated using reference solutions at pH 4 and 7 prior to the measurement.

### Free Calcium Concentration

Free calcium was quantified using the methods described by Chao et al. (2017) with modifications. Briefly, protein concentration of the exudate was determined using a Pierce BCA protein assay kit (Thermo Fisher Scientific, Waltham, MA) for use to normalize sample to sample variation of exudate. Exudate was treated with 27.5% trichloroacetic acid and centrifuged at 10,000 × *g* for 10 min. Supernatant was diluted 1:10 with ultrapure water. The diluted supernatant was filtered through a 13 mm diameter Millex-LG 0.45 μm syringe filter (Millipore, Bedford, MA), and the calcium concentrations (ppm) of the filtered samples were quantified *via* atomic absorption spectroscopy (AAnalyst 200; Perkin-Elmer, Norwalk, CT) at a wavelength of 422.7 nm with appropriate calcium concentration standards. Calcium concentrations were calculated by multiplying by the dilution factor and expressed as nmol of free calcium/mg protein.

### Sarcomere Length

Sarcomere length was determined as described by Chun et al. (2020). Briefly, pulverized PM was further homogenized in a homogenization buffer (0.25 M sucrose and 0.02 M EGTA), and ~50 μl was pipetted onto a microscope slide and sealed with a coverslip. Each sample was imaged with a Zeiss-axioplan 2 using a 100 × 1.4/f objective. Operating system used to image was LSM 5 Pascal V.3.2SP2 (Zeiss, Oberkochen, Germany). ImageJ software (version 1.52 k; National Institute of Health) with the LSM Toolbox plugin was used to analyze the images. For each sample, three images were captured and 10 sarcomeres per image were measured for an average of 30 sarcomere lengths per sample.

### Calpain Activity

Calpain activity was measured by casein zymography according to the method described by Biswas and Tandon (2019) with modifications. Briefly, sarcoplasmic proteins were mixed with Native Tris-Glycine sample buffer (Thermo Fisher) with 0.75% β-mercaptoethanol at a ratio of 1:1, and 30 μg of prepared sarcoplasmic protein samples were loaded into a resolving gel (10% acrylamide: bis-acrylamide = 37.5:1, wt/wt, containing 0.2% casein) that was polymerized with 0.72% ammonium persulfate (APS) and 0.11% tetramethylethylenediamine (TEMED) with a stacking gel (5% acrylamide, bis-acrylamide = 37.5:1, wt/wt, containing no casein) that was polymerized with 1.53% APS and 0.31% TEMED. Proteins were separated in ice-cold 1X Native-PAGE running buffer (192 mM glycine, 25 mM Tris-base, and 1 mM EDTA) with 0.05% BME using a Mini-PROTEAN Tetra System electrophoresis unit (Bio-Rad, Hercules, CA), and the system was run at a constant voltage of 125 V at 4°C until the blue tracking dye reached the bottom of the gel, approximately 4 h.

Following gel electrophoresis, gels were incubated for 1 h in 5 mM CaCl_2_ and 50 mM Tris–HCl (pH 7.5) at room temperature with one change of buffer at 30 min. Gels were incubated overnight (16 h) in 5 mM CaCl_2_, 50 mM Tris–HCl and 10 mM dithiothreitol (DTT; pH 7.5) at 25°C. After the overnight incubation, gels were stained for 30 min in Coomassie blue stain (2.3 mM Coomassie blue G250, 40% methanol, 10% glacial acetic acid, and 50% ultrapure water) at room temperature. Finally, gels were destained using a destaining solution (30% methanol, 10% glacial acetic acid, and 60% ultrapure water) at room temperature for 2 h. The gel was imaged analyzed using the ChemiDoc-It 415 Imaging System and VisionWorksLS Image Acquisition and Analysis Software (UVP, Upland, CA). Native and autolyzed forms of μ/m calpain were measured, and calpain activity was calculated by band intensities of autolyzed bands divided by band intensities of both native and autolyzed bands in a specific lane.

### Proteolysis

Degree of proteolysis was measured by troponin-T (TNT) degradation according to the method described by Chun et al. (2020) with modifications. Briefly, 60 μg of prepared myofibrillar protein samples were loaded into pre-cast RunBlue SDS 4–20% Mini Gels (BCG42012; Expedeon, San Diego, CA) and separated in ice-cold 1X RunBlue SDS running buffer (Expedeon) using a Mini-PROTEAN Tetra System electrophoresis unit (Bio-Rad, Hercules, CA). The system was run at constant voltage of 180 V until the blue dye reached the bottom of the gel (approximately 45 min). Following gel electrophoresis, gels were transferred onto a PVDF membrane using an iBlot 2 Gel Transfer Device (Thermo Fisher, Waltham, MA) with settings of 20 V for 1 min, 23 V for 4 min, and 25 V for 2 min.

Membranes were blocked and incubated overnight in mouse IgG monoclonal primary antibody (anti-TNT, JLT-12; Booster Bio, Pleasanton, CA) at a dilution of 1:20 with the blocking solution at 4°C. Following primary antibody incubation, membranes were washed and incubated in Peroxidase Conjugated Goat Anti-Mouse IgG secondary antibody (H + L; BA1050; Booster Bio) diluted at 1:1,000 with the blocking solution at room temperature while protected from light. Following the secondary antibody incubation, membranes were washed and incubated in Amersham ECL Prime Western Blotting Detection Reagent (GE Healthcare, Chicago, IL). Imaging procedure was identical as described in the *calpain activity section* using the chemiluminescent mode. Intact form of TNT was found at 35 kDa. Percent intact TNT was determined by band intensities of intact bands divided by band intensities of all bands in a specific lane.

### Collagenase Activity

Collagenase activity was measured by gelatin zymography according to the method described by Tajhya et al. (2017) with modifications. Sarcoplasmic proteins were mixed with 2X Tris-glycine SDS sample buffer (Thermo Fisher) at a ratio of 1:1. Positive control consisting of 1 nanogram of purified MMP2 [Matrix metalloproteinase-2 (MMP-2), 902-MP; R&D Systems, Minneapolis, MN] and 15 μg of prepared protein samples were loaded into a 10% Zymogram Plus (gelatin) pre-cast gel (Thermo Fisher). Proteins were separated in ice-cold 1X RunBlue SDS running buffer (Expedeon) using a Novex mini-cell XCellSure Lock electrophoresis cell (Thermo Fisher), and the system was run at constant voltage of 125 V at 4°C until the blue tracking dye reached the bottom of the gel (approximately 150 min).

Following gel electrophoresis, gels were incubated for 1 h in 1X Renaturing Buffer (Thermo Fisher) at room temperature with one change of the same buffer at 30 min. After renaturation, gels were incubated for 1 h in 1X Developing Buffer (Thermo Fisher) at room temperature with one change of the developing buffer at 30 min. Gels were incubated for 36 h in 1X Developing Buffer (Thermo Fisher) at 37°C in an incubator (Symphony general forced air incubator; VWR, Radnor, PA). After incubation, gels were stained for 30 min in Coomassie blue stain (2.3 mM Coomassie blue G250, 40% methanol, 10% glacial acetic acid, and 50% ultrapure water) at room temperature. Finally, gels were destained using a destaining solution (30% methanol, 10% glacial acetic acid, and 60% ultrapure water) at room temperature for 1 h. Imaging procedure was identical as described in the *calpain activity section*. Pro and autolyzed forms of MMP were measured, and MMP activity was calculated by band intensities of autolyzed bands divided by band intensities of both pro and autolyzed bands in a specific lane.

### Collagen Content

Collagen was hydrolyzed, and collagen content was determined by the hydroxyproline assay described by Chun et al. (2020). Briefly, pulverized PM was hydrolyzed in 6N hydrochloric acid (HCl) at 115°C for 24 h in a forced air oven. The HCl was evaporated from the samples using a vacuum evaporator (RapidVap; Labconco Corporation, Kansas City, MO) at room temperature until all residual HCl was gone. Samples were rehydrated with ultrapure water and diluted 1:400 with ultrapure water. Chloramine-T Oxidant Reagent and dimethylaminobenzaldehyde (DMBA) color reagent were added to the diluted samples and incubated in a 60°C water bath for 90 min. The samples were read at absorbance 558 nm using a spectrophotometer equipped with a microplate reader (BioTek Eon; BioTek Instruments Inc., Winooski, VT). A conversion factor of 7.14 for hydroxyproline to collagen ratio was used. Collagen content was displayed as mg of collagen per g of wet tissue.

### Collagen Crosslinks

Hydrolyzed collagen samples were purified and analyzed following methods described by Chun et al. (2020). Briefly, rehydrated samples were cleaned using a Bond Elut Cellulose cartridge 300 mg, 3 ml (12102095; Agilent Technologies, Santa Clara, CA) through a PrepSep 24-port solid-phase extraction vacuum manifold (Thermo Fisher Scientific). Cleaned samples were injected into an Acquity UPLC H-Class ultra-high-pressure liquid chromatography system (Waters Corporation, Milford, MA) equipped with a degasser, quaternary pump, sample manager and a fluorescence detector. A pyridinoline (PYD) and deoxypyridinoline (DPD) standard (*P/N* 4101; Quidel Co., San Diego, CA) was used to produce a standard curve to determine linearity range of assays and detection limits. The crosslinks were separated using an HSS T3 2.1 × 100 mm, 1.8 μm column (Waters Corporation), at a flow rate of 0.5 ml/min with a column temperature of 60°C. After a 10 min isocratic step at 100% solvent A (0.2% HFBA in ultrapure water), PYD/DPD were eluted with 85% solvent A and 15% solvent B (100% acetonitrile) for a total run time of 20 min for each sample. The PYD and DPD were measured for fluorescence at an excitation of 297 nm and emission of 395 nm. The concentration of PYD and DPD were multiplied by the dilution factors to get final concentration in ppm. To calculate the levels of crosslinks in mol/mol of collagen, the chemical masses of 428.44, 412.44, and 300,000 g/mol were used for PYD, DPD, and collagen, respectively.

### Peak Transitional Temperature Measurement of Perimysium

Perimysium extraction and denaturation temperature assessment were conducted according to methods described by Vierck et al. (2018) with modifications. Pulverized muscle tissue was homogenized in 0.05 M CaCl_2_ using a bead homogenizer (Bead Blaster 24; Benchmark Scientific, Sayreville, NJ). The homogenate was filtered through a 1 mm standard testing sieve (VWR International, Radnor, PA). The perimysial fraction collected on the screen was transferred to a microcentrifuge containing 1X PBS and hydrated for at least 1 h at 4°C prior to peak transitional temperature measurement. Ten milligram of hydrated samples was placed in a hermetic aluminum crucible (S201-53090; Shimadzu, Kyoto, Japan), and the aluminum crucible was sealed using a sealing press (SSC-30; Shimadzu). The sample crucible and a sealed empty crucible, for a reference crucible, were placed at the stage of the differential scanning calorimeter (DSC-60; Shimadzu). The temperature program was set from room temperature to 100°C with a 5°C/min temperature increase. To analyze the data the TA-60WS software (Shimadzu) was used to determine the peak transitional temperature (°C).

### Sarcoplasmic Reticulum Extraction

The SR membrane was extracted from muscle tissue according to the method described by Wilkie and Schirmer (2008) with modifications. Briefly, prefabricated PM strip was cut into small 1 cm × 1 cm × 1 cm cubes and homogenized in ice-cold homogenization buffer (50 mM HEPES, 24 mM KCl, 5 mM MgCl_2_, and 250 mM sucrose with 0.1% Halt Protease Inhibitor Sing-Use Cocktail; Thermo Scientific, Waltham, MA). Content was centrifuged at 1000 × *g* for 10 min at 4°C to remove myofibrillar proteins, centrifuged at 10,000 × *g* for 20 min at 4°C to pellet the mitochondria, and centrifuged at 100,000 × *g* for 45 min at 4°C (Optima XE-90 Ultracentrifuge, Type 45 Ti rotor; Beckman Coulter) to pellet the microsome containing the SR. The supernatant was discarded, and the pellet was resuspended in ice-cold homogenization buffer, 2.8 M SHKM buffer (2.8 M sucrose, 50 mM HEPES, 25 mM KCl, and 5 mM MgCl_2_) and 1.85 M SHKM (1.85 M sucrose, 50 mM HEPES, 25 mM KCl and 5 mM MgCl_2_) at the ratio of 10:19:7, respectively. The samples were centrifuged at 57,000 × *g* for 4 h at 4°C (SW32 Ti rotor; Beckman Coulter) using the principle of density gradient centrifugation to move the less dense SR to the layer with less sucrose while pelleting the denser components of the microsome. The SR was removed from the interphase between the 1.85 M SHKM and homogenization buffer and resuspended with ice-cold homogenization buffer. The SR sample was centrifuged one last time at 152,000 × *g* for 75 min at 4°C (SW32 Ti rotor; Beckman Coulter). The supernatant was discarded, and the pellet was resuspended in homogenization buffer. Half of it was used for proteomic analysis, and the other half was used for lipidomics analysis.

### Protein Extraction

Microcentrifuge tubes containing the SR proteomic samples were centrifuged at 6,000 × *g* for 10 min at 4°C to pellet the SR. The supernatant was discarded, and the pellet was resuspended and vortexed in 100 μl 1 X PBS containing 0.1% Triton X-100 and 150 μl of 8 M urea. The samples were further centrifuged at 6,000 × *g* for 2 min at 4°C to re-pellet the insoluble material. The supernatant was collected, the protein concentration was determined using a Pierce BCA protein assay kit (Thermo Fisher Scientific). The protein stock was stored at -80°C until further analysis.

### Shotgun Proteomics

Shotgun proteomics were conducted in Center for Genomics and Proteomics at Oklahoma State University. The SR protein stock containing 50 μg of protein was reduced with the addition of 5 mM tris (2-carboxyethyl) phosphine for 30 min at room temperature. The reduced samples were further alkylated for 30 min in the dark at room temperature by the addition of 10 mM iodoacetamide. The solutions were diluted with three volumes of 100 mM Tris–HCl at pH 8.5 and digested by the addition of one μg/mL of trypsin/LysC (Promega, Madison, WI). After overnight digestion, an additional 0.5 μg/ml of trypsin/LysC were added and digested for another 6 h. Peptides were desalted by solid-phase extraction using a C18 pipet tip (Pierce 87784; Thermo Fisher Scientific) and eluted with 70:30:0.1 acetonitrile/water/trifluoroacetic acid.

Peptides were injected into an Acclaim PepMap RSLC nano-C18 column (2 μm C18 particles, 75 μm ID × 50 cm, Thermo Fisher) in a vented trap configuration and separated using a gradient of 0.1% formic acid/acetonitrile (3–30%) for 120 min. The peptides were eluted into a Nanospray Flex ion source coupled to an Orbitrap Fusion mass spectrometer (Thermo Fisher Scientific). The mass spectrometer was programmed to perform “Top-Speed” data-dependent MS/MS using quadrupole filtration, HCD collision, and ion trap analysis of fragment ions.

Instrument RAW files were analyzed using MaxQuant v1.6.10.43 to compare observed ions of peptides and peptide fragments to a database of 27,542 Gallus gallus sequences downloaded from Uniprot on 01/19/20. The database searches utilized default MaxQuant settings, supplemented with the variable following modifications: oxidation of Met, N-terminal acetylation, and cyclization of Gln to pyro-glutamate.

### Lipid Extraction and Preparation

The SR lipid was extracted according to the method described by Chao et al. (2020) with modifications. Briefly, SR stock was mixed with 1:2 chloroform: methanol (v/v) and incubated overnight. The next morning, chloroform and ultrapure water were added, shaken, and centrifuged, and the bottom layer was transferred and dried under nitrogen. Finally, 1 ml of chloroform was added to redissolve the extracted lipid and stored at −80°C until sample preparation. A predetermined volume of lipid stock in chloroform corresponding to approximately 2.5 μl diluted lipid in chloroform per mg of protein, was transferred to an amber GC vial. Precise amounts of phospholipid internal standards were added to each vial, and a solvent (chloroform: methanol: 300 mM ammonium acetate in water, 300:665:35, v/v/v) was added to the sample.

### Electrospray Ionization-Triple Quadrupole Mass Spectrometry

Lipidomics analysis of prepared lipid samples were analyzed at the Kansas Lipidomic Research Center (Kansas State University) following the methods described in detail by Chao et al. (2020). Each sample underwent a precursor and neutral loss scan to create a set of spectra with each spectrum exposing a set of lipid species containing a common head group or fatty acids fragment. The parameters used to detect phospholipid species were as described by Xiao et al. (2010) particularly the intact ion analyzed, fragment type, scan mode, polarity, collision energy, declustering potentials, entrance potentials, and exit potentials. Within each phospholipid class, the lipid peak areas were uploaded to an online processing software for direct-infusion mass spectral data for lipid profile—LipidomDB Data Calculation Environment (Zhou et al., 2011). Data were conveyed as mole percent (mol%)—each phospholipid species in relative % of total phospholipid. This was accomplished by multiplying each nanomolar value by 100% and dividing by the total of the nanomolar amounts of the lipids analyzed. Finally, each evident lipid molecular species is exhibited as total acyl carbons: total double bonds. For example, 38:3 in PC represents 38 acyl carbons with 3 double bonds.

### Statistical Analysis

The pH, sarcomere length, free calcium content, calpain activity, TNT degradation, collagenase activity, collagen content, collagen crosslink density, connective tissue peak transitional temperature, and lipidomics data were analyzed as a completely randomized design. Each animal was considered the experimental unit. Data were analyzed using the GLIMMIX procedure of SAS (version 9.4, Cary, NC). For the lipidomic data, which included 352 phospholipid species identified, a SAS macro was constructed to automatically conduct the analyses repeatedly for all 352 identified phospholipid species. Separation of means was conducted using LSMEANS procedure (least significant differences) at *p* ≤ 0.05.

The Perseus software (v1.6.1.1) was used to analyze the label-free quantification (LFQ) protein intensities data files to perform t-tests with a random permutation false discovery rate correction (FDR) set to 0.05 with an S0 factor of 0.1 to determine proteins with significant expression (*p ≤* 0.05). Differences between protein intensities were validated with log_2_ transformed LQF protein intensities. Following statistical analysis, DAVID (v6.8)^1^ software was utilized to classify gene ontology enrichment analysis of the functional classes of the significantly abundant proteins.

## Results and Discussion

### pH Determination

The pH values for WB were higher compared to the N samples (6.17 vs. 5.83; *p <* 0.01; Table 1). This is supported by many other studies (Mudalal et al., 2015; Cai et al., 2018; Baldi et al., 2020), who also found elevated pH in the WB fillets. While the actual cause of the pH increase in WB is not known, there are several hypotheses. Kuttappan et al. (2017) suggested that there is a downregulation of carbohydrate metabolism in WB, which would lead to less lactate being produced during the rigor mortis process and result in higher ultimate pH. On the other hand, Mudalal et al. (2015) suggested the presence of excessive scar tissue may lower the glycogen content in WB PM samples, resulting in a higher ultimate pH. However, more research would be needed to confirm these hypotheses.

**Table 1:** Effects of woody breast (WB) myopathy on pH, free calcium concentration, sarcomere length, calpain activity, proteolysis, collagenase activity, collagen content, pyridinoline (PYD) density, deoxypyridinoline (DPD) density and connective tissue peak transitional temperature compared to normal (N) Pectoralis major from broilers.

Measurements	WB	N	SEM	Value of *p*
pH	6.17	5.83	0.04	<0.01
Free Calcium Concentration, nmol calcium/mg protein	6.23	4.17	0.60	<0.05
Sarcomere Length, μm	1.70	2.02	0.10	0.05
Calpain Activity, % calpain autolyzed	71.05	59.12	2.10	<0.01
Proteolysis, relative % intact troponin-T	49.98	56.97	2.26	0.05
Collagenase Activity, % activated MMP	13.24	7.84	1.43	<0.05
Collagen Content, mg collagen/g wet tissue	3.89	2.08	0.34	<0.01
PYD Density, mol PYD/mol collagen	0.23	0.14	0.02	<0.05
DPD Density, mol DPD/mol collagen	0.07	0.04	0.007	<0.01
Peak Transitional Temperature, °C	65.47	63.72	0.53	<0.05

### Free Calcium Concentration

Exudate from WB had greater free calcium concentration compared to those from N PM (6.23 vs. 4.17 nmol calcium/mg protein; *p <* 0.05; Table 1). Many other studies have also found higher free calcium concentrations in the WB samples (Soglia et al., 2016, 2018; Tasoniero et al., 2016). Calcium plays an important role in rigor mortis as well as the following tenderization process as many proteolytic enzymes require calcium for activation (Koohmaraie and Geesink, 2006). However, the exact cause for the increase in free calcium in WB samples is not fully understood. One of the hypotheses suggested that the calcium sequestering capabilities of the SR may be compromised in WB broilers (Soglia et al., 2018). As mentioned in the introduction, SR’s number one role is to maintain calcium homeostasis within the cells to ensure the proper balance between muscle contraction and relaxation (Barone et al., 2015). Damages in SR proteins can result in higher levels of free calcium leakage into the sarcoplasm. Additionally, Mutryn et al. (2015) noted an upregulation of the gene *PLA2G4A* which encodes for PLA2 in broilers, which suggested that extra sarcoplasmic calcium may activate PLA2 resulting weakening of membrane from various locations.

### Sarcomere Length

**Figure 2:**
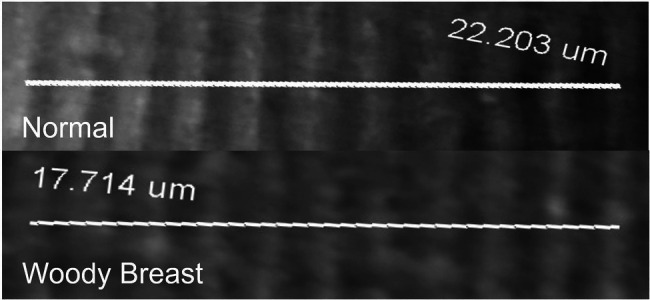
Representative images of Woody Breast (WB) and normal (N) Pectoralis Major muscle fiber used to quantify sarcomere length.

Sarcomere length was shorter in WB compared to N PM (1.70 vs. 2.02 μm; *p =* 0.05; Table 1). A representative image for sarcomere length measurements is displayed in Figure 2. It is interesting to note that many past studies observed longer sarcomeres in the WB compared to N PM (Solo, 2016; Tijare et al., 2016; Sun et al., 2018). The variation of deboning and freezing times as well as the WB severity could explain some of the differences observed. The samples in the current study were deboned at 3 h postmortem and frozen around 8 h, whereas Tijare et al. (2016) deboned at 4 h postmortem and frozen around 24 h. In addition, Solo (2016) deboned and immediately froze their samples at 4 h postmortem, and Sun et al. (2018) deboned at 2 h postmortem and only utilized chilled storage for 1, 2, and 8 d. In poultry, the rate of rigor mortis can vary depending on various factors both pre- and postmortem but may take as long as 6 h to complete (Schreurs, 2000). Tasoniero et al. (2020) found that deboning time significantly affected the differences in texture measurements between cooked N and WB PM. In addition, it was also demonstrated that the differences in sarcomere lengths could be affected by processing treatments or the sampling location in PM (Puolanne et al., 2021). During the rigor mortis process, free calcium plays a critical role in the formation of actomyosin bonds and the ultimate sarcomere length (Ertbjerg and Puolanne, 2017). Our calcium data supported the shorter sarcomere phenomenon observed in this study as it is possible that the higher free calcium in WB samples allowed for the additional development of actomyosin complexes during the rigor mortis process and resulted in the observed phenomenon for shorter sarcomere lengths in WB. However, Liu et al. (2020) has also noted that calcium sensitivity was decreased in WB PM. Therefore, it is still unclear the true reason for the observed shorter sarcomeres in WB PM for this study.

### Calpain Activity and Proteolysis

**Figure 3:**
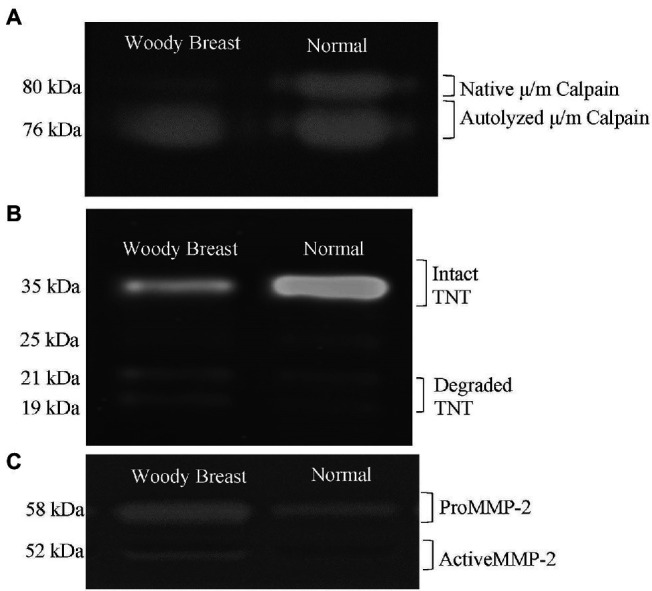
Representative images of Woody Breast (WB) and normal (N) Pectoralis Major muscle proteins used to quantify **(A)** calpain activity; **(B)** troponin-T (TNT) proteolysis; **(C)** matrix metalloproteinase (MMP) activity.

At 8 h postmortem, more μ/m calpain was autolyzed in WB samples compared to the N samples, indicating greater calpain activity in WB than N PM (71.05 vs. 59.12% calpain autolyzed; *p <* 0.01; Table 1). A representative image for the calpain activity measurement is displayed in Figure 3A. In addition, WB samples had less intact TNT compared to N samples (49.98 vs. 56.97% of intact TNT; *p =* 0.05; Table 1). A representative image for the TNT degradation measurement is displayed in Figure 3B. Calpain autolysis has been shown to have a positive relationship to storage time and myofibrillar protein degradation (Koohmaraie and Geesink, 2006). Soglia et al. (2018) observed an increase in autolyzed μ/m calpain simultaneously with a decrease in native μ/m calpain in both the WB and N samples as they were stored for 10, 24, 72, 120 and 168 h postmortem at 5°C. Hasegawa et al. (2020) also found WB samples to have higher calpain activity and less intact TNT measured at 12 h postmortem compared to the N samples. Furthermore, Bowker et al. (2016) reported 24 h postmortem WB PM to have less intact desmin compared to N samples. It is important to point out that μ/m calpain has been shown to be the dominant calpain form in avian muscles (Soglia et al., 2018), while μ-calpain become inactive within 6 h postmortem (Lee et al., 2008). Therefore, it is very likely that the calpain activity identified through the casein zymography in the current study was from μ/m calpain. Finally, the calpain system is calcium- and pH-dependent (Koohmaraie and Geesink, 2006), so it is possible the increased free calcium available in WB samples as well as the great ultimate pH observed in this study contributed to the enhanced proteolytic activity.

### Collagenase Activity

The WB samples had greater relative percentage of activated MMP demonstrating greater collagenase activity compared to the N samples (13.24 vs. 7.84% activated MMP; *p <* 0.05; Table 1). A representative image for the collagenase activity measured using gelatin zymography is displayed in Figure 3C. Collagenase MMPs are a group of calcium-dependent proteases that degrade components of the extracellular matrix, such as collagen and gelatin (Visse and Nagase, 2003). It is possible that the collagenase MMP activity is also enhanced in WB samples due to the increased levels of free calcium found in this study. Byron et al. (2019) found that visual WB defects, such as the hard rigid appearance began to dissipate, and the flexibility of the fillet improved after refrigerated storage of 5 days, which mitigate the severity of WB. It is possible that the enhanced collagenase activity in WB samples may play a role in the dissipation of WB during chilled storage. However, there is currently too little research in collagenase activity of WB PM to make any concrete conclusions.

### Collagen Content and Crosslink Density

The WB had more collagen content compared to N samples (3.89 vs. 2.08 mg collagen/g wet tissue; *p <* 0.01; Table 1). In addition, WB samples had greater PYD and DPD crosslinks density than the N samples (0.23 vs. 0.14 mol PYD/mol collagen and 0.07 vs. 0.04 mol DPD/mol collagen, respectively; *p <* 0.05; Table 1). Many others have also found WB PM to have more intramuscular collagen compared to N samples (Soglia et al., 2016; Baldi et al., 2019). Baldi et al. (2019) attributed the cause of the increased collagen content in WB samples as progressive myodegeneration, which results in the deposit of scar tissue instead of muscle fibers. Both Sihvo et al. (2014) and Soglia et al. (2016) also suggested that fibrosis may also be occurring in WB broilers, which leads to the thickening of connective tissue throughout the PM sample. On the other hand, Baldi et al. (2019) also showed that WB samples had greater PYD density than the N samples. Observing the development of scar tissue and constrictive remodeling of arterial tissue in rabbits has been shown to impact excessive crosslinking (Brasselet et al., 2005). Therefore, the scar tissue and fibrosis in WB filets could potentially be leading to the promotion of high densities of collagen crosslinking. Finally, Wu et al. (2021) documented a strong positive correlation between PYD density in raw beef shank cuts and WBSF and connective tissue texture evaluated by East Asian consumers. It is likely the increase in mature collagen crosslink density may contribute to the rubbery texture and increased thermal denaturation temperature of the connective tissue observed in WB samples from this study.

### Peak Transitional Temperature of Perimysium

The WB samples had a greater collagen denaturation temperature compared to N samples (65.47 vs. 63.72°C; *p <* 0.05; Table 1). Baldi et al. (2019) associated the peak at 60.4 ± 1.5°C as the collagen denaturation temperature in poultry meat. Torrescano et al. (2003) associated higher transitional temperature reading for beef muscles with greater shear force, and many studies have shown that the peak transitional temperature of connective tissue is an indicator of greater concentration of thermally stable collagen crosslinks (Horgan et al., 1990; Tornberg, 2005), which agreed with our finding in this study.

### Proteomics

**Figure 4:**
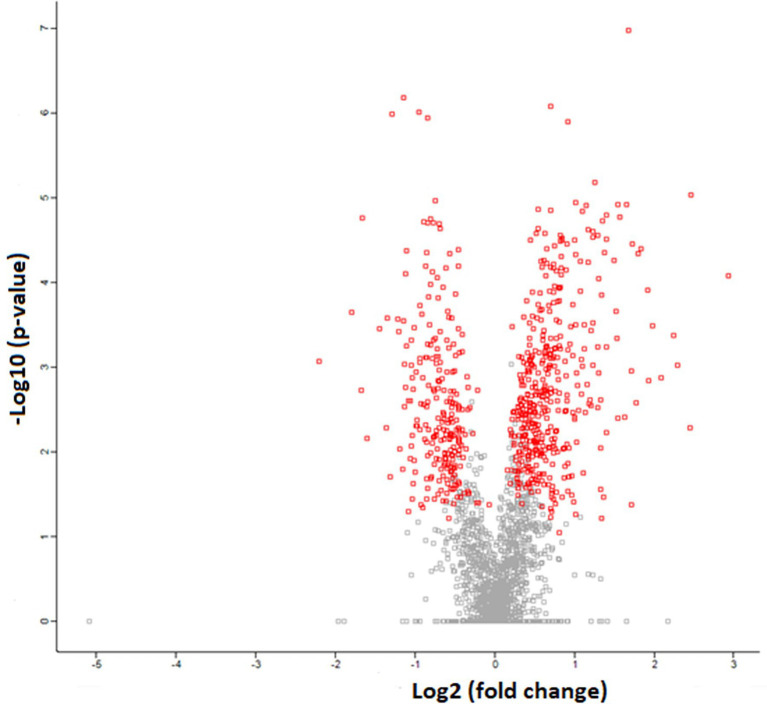
Volcanic plot representation of Woody Breast and Normal sarcoplasmic reticulum proteome utilizing high-stringency criteria (FDR 0.05 and S0 factor 0.1) in which red dots correspond to significantly upregulated or downregulated proteins (*p* < 0.05). The gray dots represent proteins that are not different (*p* > 0.05).

The SR proteomics data identified over 2,700 proteins, with 677 proteins having significant differential expression (*p* < 0.05) between WB and N samples. Of those proteins demonstrating differential expression, 431 were upregulated and 246 proteins were downregulated in WB samples compared to N as demonstrated by the volcano plot where the red dots represent significantly different proteins and the gray dots represent proteins not significantly different (Figure 4). The proteomics results are listed as Log2 fold changes, where a positive value indicates more expression in WB and a negative value more expression in N. Log2 fold change can be converted to fold change by taking 2 to the power of the Log2 fold change value. As demonstrated in past SR proteomic studies (Babu et al., 2004; Liu et al., 2013), our SR proteome coverage goes way beyond proteins involved in calcium homeostasis. In order to achieve our objective, this paper will focus on discussing the findings related to proteins involved in calcium regulation, membrane integrity, acetylcholinesterase activity, and stress response (Table 2).

**Table 2:** Representative proteins related to the calcium regulation, membrane integrity, acetylcholinesterase activity, endoplasmic reticulum (ER) stress response pathways in woody breast (WB) and normal (N) pectoralis major sarcoplasmic reticulum.

Protein name	Gene name	UniProt ID	Value of *p*	Log2 fold changes (WB/N)	Specific function
Calcium regulation
Sarcoplasmic/endoplasmic reticulum calcium ATPase 2	SERCA	Q03669	<0.01	1.68	Calcium transport
Calcium-transporting ATPase	ATP2B4	A0A1D5PU07	<0.05	1.37	Calcium transport
Plasma membrane calcium-transporting ATPase 1	ATP2B1	Q98SH2	<0.01	0.73	Calcium transport
Ryanodine receptor 2	RYR2	F1NLZ9	<0.01	−0.98	Calcium release
Ryanodine receptor 3	RYR3	A0A1D5PCL0	<0.01	−0.64	Calcium release
Calcium load-activated calcium channel	TMCO1	A0A1L1RIN1	<0.05	−0.64	Calcium release
Voltage-dependent L-type calcium channel subunit alpha	CACNA1S	A0A1D5PTJ2	<0.05	−0.52	Calcium release
Junctophilin 1	JPH1	A0A1D5P1R0	<0.01	−0.59	Calcium release
Calsequestrin	CASQ2	A0A3Q2TXF6	0.30	−0.28	Calcium storage
Calmodulin	CALM	P62149	0.33	−0.28	Calcium storage
Sarcalumenin	SRL	A0A1D5P984	<0.01	1.09	Calcium storage
Membrane integrity
Phospholipase A2	PLA2G4A	A0A1D5PS91	<0.01	0.75	Enzymatic fatty acid cleavage
Annexin A1	ANXA1	F1N9S7	<0.01	0.84	Phospholipase A2 suppressor
Annexin A2	ANXA2	P17785	<0.01	1.17	Phospholipase A2 suppressor
Annexin A4	ANXA4	A0A1D5PTL7	<0.01	1.41	Phospholipase A2 suppressor
Annexin A5	ANXA5	P17153	<0.01	0.69	Phospholipase A2 suppressor
Annexin A6	ANXA6	P51901	<0.01	1.80	Plasma membrane repair
Myoferlin	MYOF	A0A1D5NT41	<0.01	1.22	Plasma membrane repair
Acetylcholinesterase activity
Cholinesterase	BCHE	A0A3Q2UC01	<0.01	2.93	Acetylcholinesterase activity
Guanine nucleotide binding protein G11 alpha-subunit	GNA11	Q71RI7	<0.01	0.66	G protein-coupled acetylcholine receptor signaling pathway
Guanine nucleotide binding protein, q polypeptide	GNAQ	Q5F3B5	<0.01	0.45	G protein-coupled acetylcholine receptor signaling pathway
Synaptophysin-like protein 2	SYPL2	A0A3Q2UCF9	<0.05	−0.59	Calcium homeostasis
ER stress response
Protein disulfide-isomerase	P4HB	A0A1D5PV06	<0.01	0.51	Response to ER stress
Protein disulfide-isomerase A3	PDIA3	Q8JG64	<0.05	0.53	Response to ER stress
Protein disulfide-isomerase A4	PDIA4	A0A1D5PWP7	<0.01	0.40	Response to ER stress
Thioredoxin-related transmembrane protein 4	TMX4	R4GFY2	<0.01	0.52	Response to ER stress

*The proteomics results are listed as Log2 fold changes, where a positive value indicates more expression in WB and a negative value more expression in N*.

#### Proteins Associated With Calcium Regulation

The first part of Table 2 provides a cluster of known proteins associated with SR that play significant roles in calcium regulation. Calcium transport proteins like SERCA, calcium-transporting ATPase, and plasma membrane calcium-transporting ATPase 1 were upregulated in WB compared to N samples (*p <* 0.05). On the other hand, calcium-release proteins like RYR 2 and 3, calcium load-activated calcium channel, and Junctophilin 1 were downregulated in WB samples, (*p <* 0.05). Finally, no differences were found between WB and N for major calcium storage proteins, such as CASQ and calmodulin (*p >* 0.05). However, an upregulation of sarcalumenin was observed for the WB samples compared to N (*p <* 0.01), which also serves a calcium storage function in striated muscle (Papah et al., 2018).

There has been ample research linking calcium level imbalance to myopathies (Chelu et al., 2004; Bellinger et al., 2008). SERCA and other calcium transport proteins are responsible for the rapid removal of calcium from the sarcoplasm to induce muscle relaxation and to replenish calcium stores within the cell (Periasamy and Kalyanasundaram, 2007). Morel et al. (2004) documented an upregulation of SERCA and a downregulation of RYRs in muscular dystrophy mice. In addition, Soglia et al. (2016) and Mutryn et al. (2015) also observed a greater relative abundance of SERCA and calcium-transporting ATPase in WB compared to N samples and discussed the possibility of WB muscle cells increased calcium transport protein synthesis in response to increased amount of sarcoplasmic calcium. Calcium-release proteins like RYR are embedded in the plasma membrane of SR and are responsible for the release of calcium from the SR during excitation–contraction coupling (Lanner et al., 2010). Marchesi et al. (2019) also found that RYR2 and 3 genes were downregulated in WB samples through transcriptomic analysis and discussed the likelihood of high sarcoplasmic calcium may be triggering necrosis of muscle cells in WB broilers. Finally, myopathies, such as Duchenne muscular dystrophy, has shown to reduce the expression of calcium storage proteins (Doran et al., 2004). However, no consistent modifications in calcium storage proteins were observed in the current study between WB and N samples. These results suggested that the WB SR may be responding to the extra calcium in the sarcoplasm by increasing the protein synthesis of calcium transport proteins while decreasing the protein synthesis of calcium-release proteins to maintain calcium homeostasis. The calcium storage capabilities of the muscle were likely not altered by the WB myopathy.

#### Proteins Associated With Membrane Integrity

Table 2 also provides a list of proteins found in this study that are related to the membrane integrity of the SR. The membrane of the SR is made up of a phospholipid bilayer in which multiple proteins are embedded, particularly those involved in translocating calcium (Zhang et al., 1997). The PLA2 can hydrolyze the phospholipids into lysophospholipids and result in significant damage to the membranes (Diaz and Arm, 2003). In this study, we observed an upregulation of PLA2 in WB compared to the N samples (*p <* 0.01). Many studies found that PLA2 was upregulated due to the activation of mitogen-activate protein kinase cascades, which is linked to a wide range of extracellular stimuli such hormones, calcium mobilizing agents, oxidative stress, or osmotic challenges (Lambert et al., 2006; Rubio et al., 2015). Furthermore, Greene et al. (2020) and Xing et al. (2021) both noted an upregulation of PLA2 in WB compared to the N samples, and they have linked this observation with increased production of reactive oxygen species in WB broilers. Additionally, multiple PLA2 suppressor proteins such annexin A1, A2, A4, and A5 were significantly upregulated in the WB samples (*p <* 0.01). These proteins are responsible for inhibiting PLA2 activity (Kim et al., 1994), which further confirmed our findings on the overexpression of PLA2 in this study.

Annexin A6 and myoferlin, proteins involved in plasma membrane repair were also significantly upregulated in WB compared to N samples (*p <* 0.01). Annexin A6 is a protein that is often recruited after membrane disruption and has been noted to be part of the plasma membrane repair system (Boye and Nylandsted, 2016). On the other hand, myoferlin is responsible for aiding in membrane repair specifically *via* plasma membrane fusion during regeneration (Cipta and Patel, 2009). Overall, the results imply that the SR membrane is undergoing some level of damage from PLA2 hydrolysis. Regulatory proteins are attempting to inhibit PLA2 activity, while the upregulation of membrane repair proteins demonstrated the cells’ effort to restore SR membrane integrity. However, it is possible that SR membrane has sustained enough damage from PLA2 hydrolysis, which could lead to calcium leakage from the SR.

#### Proteins Associated With Acetylcholinesterase Activity

The next part of Table 2 provides a list of key proteins involved in acetylcholine esterase activity. One specific protein, cholinesterase, had almost an eight-fold increase in WB compared to N (*p <* 0.01), and this protein exhibited the highest fold change of all the proteomics data collected for this study. Cholinesterase is responsible for the breakdown of acetylcholine into choline and acetic acid (Quinn, 1987). The hydrolysis of acetylcholine plays a key role in muscle contraction as it terminates the action potential and begins the membrane repolarization process, which allows for the relaxation of muscle (Colovic et al., 2013). Upregulation of cholinesterase is often associated with neurodegenerative disorders, such as dementia (Yang et al., 2020), Alzheimer’s (McGleenon et al., 1999), and Parkinson’s disease (Ruberg et al., 1986). Multiple studies have also observed increased cholinesterase expression due to a disturbance in calcium homeostasis, particularly with elevated sarcoplasmic calcium levels (Bursztajn et al., 1991; Sberna et al., 1997). This coincides with the finding from the current study as WB is known to have higher levels of sarcoplasmic calcium compared to N samples. Additionally, there are many reversible/irreversible cholinesterase inhibitors, such as organophosphorus insecticides, that can also result in the dramatic upregulation of cholinesterase (Fulton and Key, 2001). With such unique data found in this study, we hypothesize there may be cholinesterase inhibitors present within the system, resulting in the increase production of cholinesterase in WB broilers to prevent the prolonged calcium release from the SR, which results in muscle contraction.

There is also a significant upregulation of guanine nucleotide binding protein (G protein) G11 alpha-subunit and G protein q polypeptide in WB samples (*p <* 0.01). These proteins are involved in the G protein-coupled acetylcholine receptor signaling pathways by binding guanosine triphosphate to induce a conformational change and activation of phospholipase C (Putney and Tomita, 2012). This chain reaction signals SR to release calcium resulting in elevating the sarcoplasmic calcium level (Alvarez-Curto et al., 2016). Wagner et al. (2010) found that knockout G protein G11 alpha-subunit and G protein q polypeptide mice had shorter action potential compared to the wild type. Therefore, the observed upregulation of G protein-coupled acetylcholine receptor signaling pathway could translate into prolonged action potential leading to potentially more calcium release from the SR. On the other hand, synaptophysin-like protein 2 (SYPL2), was found to be downregulated in WB compared to the N samples (*p <* 0.05). The main function of this protein is to maintain calcium homeostasis as it controls calcium release *via* communication between the T-tubule and the junctional SR membrane (Kurebayashi et al., 2003). Papah et al. (2018) also noted a downregulation of SYPL2 in WB samples and linked this phenomenon to excitation–contraction coupling disruption and alteration of calcium homeostasis. In combination with the downregulation of RYR found in this study, it further confirms our earlier speculation that the WB SR is attempting to restore calcium homeostasis by downregulating calcium-release channels from the SR.

#### Proteins Associated With ER Stress

Finally, Table 2 provides a list of proteins found in this study that are related to endoplasmic reticulum (ER) stress response. The SR is a specialized form of the ER that only exists in the muscle tissue (Villa et al., 1993). Protein disulfide-isomerase, protein disulfide-isomerase A3 protein disulfide-isomerase A4, and thioredoxin-related transmembrane protein 4 were upregulated in the WB compared to N samples (*p <* 0.05). Mutryn et al. (2015) demonstrated that WB tissues are in hypoxic state from poor vascularization, and Abasht et al. (2016) showed that WB broilers exhibited oxidative stress. Inagi (2010) concluded that the increased expression of the ER stress proteins can be attributed to hypoxia or oxidative stress. When ER/SR endures stress, protein misfolding and aggregation of misfolded proteins have been documented to result in diseases, such as cancer and neurodegeneration (Rao and Bredesen, 2004). Fortunately, the ER/SR has various stress response pathways that can be activated to combat protein misfolding. Protein disulfide isomerases combat ER/SR stress by rearranging and replacing disulfide bonds within misfolded proteins to correct the folding error (Gilbert, 1997), and thioredoxin-related transmembrane protein 4 provides necessary quality control for ER/SR protein folding (Roth et al., 2010).

### Lipidomics

**Figure 5:**
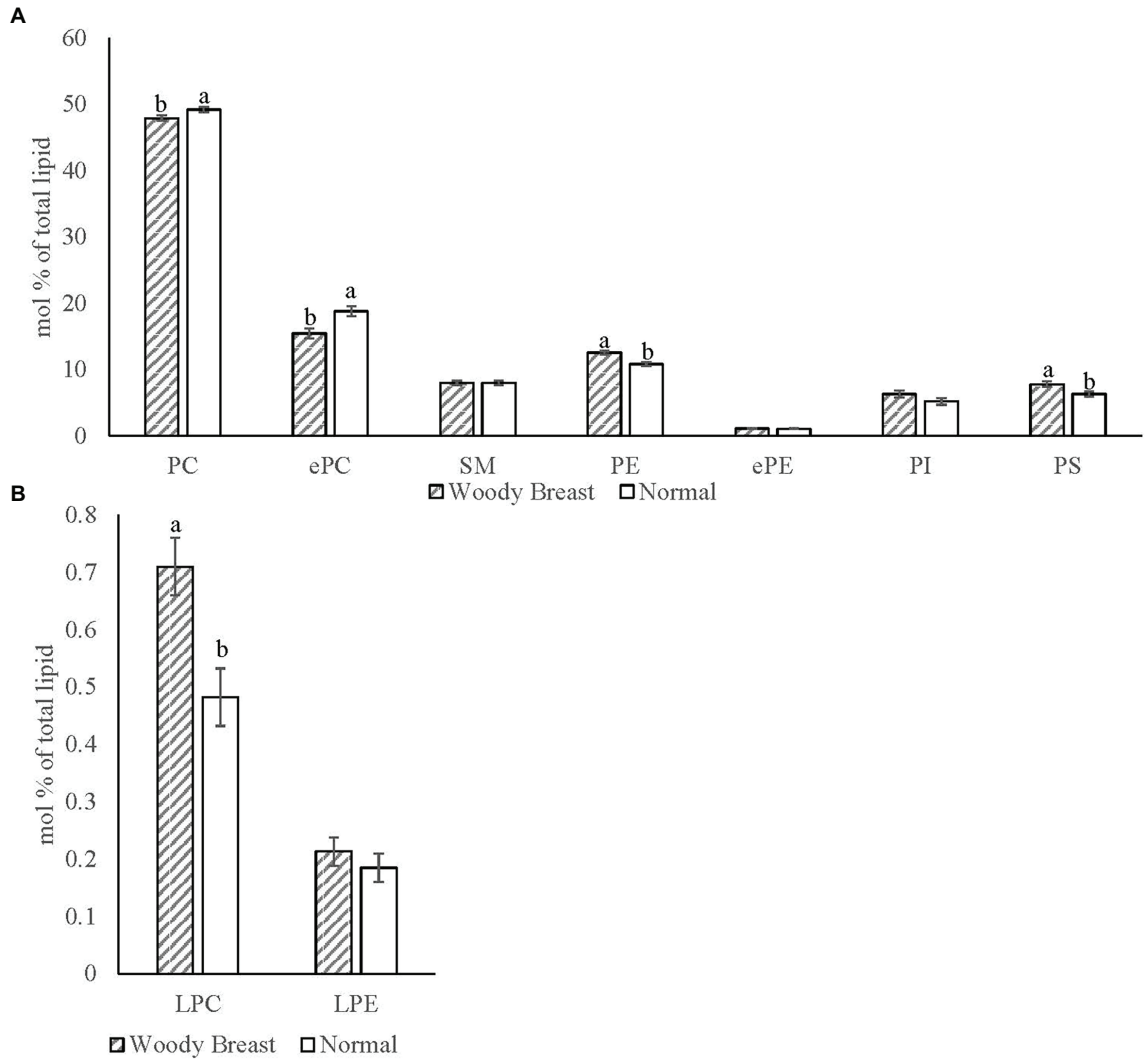
Woody breast syndrome altered sarcoplasmic reticulum **(A)** phospholipid classes and **(B)** hydrolysis product mol% [(nmol of phospholipid class/nmol of total phospholipid) x100]. Each bar represents the mean ± standard error and means with different letters within a lipid class are different at *p* < 0.05. PC, phosphatidylcholine; ePC, ether-linked PC; SM, sphingomyelin; PE, phosphatidylethanolamine; ePE, ether-linked PE; PI, phosphatidylinositol; PS, phosphatidylserine; LPC, lysophosphatidylcholine; LPE, lysophosphatidylethanolamine.

The Electrospray Ionization (ESI) MS/MS characterized 352 different polar lipid species in chicken breast SR with the major phospholipid classes identified being LPC, PC, ePC, SM, LPE, PE, ePE, PI, and PS. Sixty-six of those identified lipid species were significantly different (*p* < 0.05) between WB and N samples. Figure 5 displays the mol% of the SR (A) phospholipid classes and (B) hydrolysis products for WB and N samples. There was less PC and ePC and more PE and PS in WB compared to N SR (*p <* 0.05). In addition, WB SR had significantly more total LPC compared to N samples (*p <* 0.05).

Table 3 shows the major individual phospholipid molecular species mol% that demonstrated statistical differences (*p <* 0.05). The decrease of PC mol% in WB SR is largely due to the decrease of PC 34:1, 36:3, 36:4, and 38:5 (*p <* 0.05). The ePC mol% followed a similar trend as the PC, in which mol% for ePC 36:4, 36:5, 38:4, 38:5, and 38:6 were reduced for WB SR compared to N (*p <* 0.01). For both PC and ePC, there was in increase in mol% for 32:0 for WB SR (*p <* 0.05). Elevated levels of PE in WB SR is due to the mol% increase in PE 34:1, 36:1, 36:2 and 40:4 (*p <* 0.05). Finally, WB SR had greater LPC mainly due to an increase in mol% for LPC 16:0 and 18:1 (*p <* 0.05).

**Table 3:** Representative phospholipid molecular species (total acyl carbons: total double bonds) mol% of phosphatidylcholine (PC), ether-linked PC (ePC), phosphatidylethanolamine (PE), and LysoPC (LPC) in woody breast (WB) and normal (N) pectoralis major sarcoplasmic reticulum.

	WB	N	SEM	Value of *p*
PC, %
32:0	3.73	2.52	0.28	<0.05
34:1	14.00	15.57	0.29	<0.01
36:3	2.45	2.82	0.11	<0.05
36:4	2.31	2.53	0.06	<0.05
38:3	0.67	0.58	0.02	<0.05
38:5	0.69	0.80	0.02	<0.01
ePC, %
32:0	0.64	0.44	0.04	<0.01
36:4	1.85	2.42	0.10	<0.01
36:5	1.91	2.62	0.08	<0.01
38:4	0.97	1.38	0.06	<0.01
38:5	1.51	2.06	0.07	<0.01
38:6	0.77	1.10	0.04	<0.01
PE, %
34:1	0.85	0.74	0.03	<0.05
36:1	1.47	0.95	0.05	<0.01
36:2	2.74	2.29	0.08	<0.01
40:4	0.72	0.53	0.04	<0.05
LPC, %
16:0	0.23	0.14	0.02	<0.05
18:1	0.15	0.11	0.01	<0.01

The PC is the major phospholipid class and the main building block of the mammalian cell membrane (Kanno et al., 2007). The PC can be further divided into two groups. Typical PC contains an ester bond between the glycerol backbone and fatty acid side chains, and the ePC which contains an ether bond instead of ester bond (da Silva et al., 2012). Vu et al. (2015) found that lipid bilayers are particularly sensitive to hydrolysis by the PLA2 when the cells are undergoing stress response, which is supported by our finding that PLA2 was upregulated in WB compared to the N samples. Mouchlis et al. (2018) found that PLA2 exhibited greater activity toward PC over other phospholipid classes, which likely explained the decrease in both PC and ePC classes, but not in other phospholipid classes in WB samples. Dorninger et al. (2020) suggested ether-linked phospholipids may have the ability to influence signal transduction pathways by altering the function of plasma membrane receptors. Perhaps, the reduction of ePC for WB samples is an indication of dysfunctionality in the signaling cascades, which resulted in the upregulation of proteins participated in the G protein-coupled acetylcholine receptor signaling pathways in WB samples as mentioned earlier. Finally, Chun et al. (2021) found similar phospholipid alteration results in an *in vitro* liposome study utilizing PLA2, which they also found a decrease in molecular species, such as PC/ePC 36:3, 36:4, and 36:5 due to PLA2 hydrolysis.

The second most abundant phospholipid in mammalian cells is PE (Vance, 2015), and this phospholipid class serves to stabilize membrane proteins and protein complexes within the lipid bilayers (Farine and Bütikofer, 2013). Many studies have found a high PC:PE ratio contributes to reduced SERCA activity (van der Veen et al., 2017; Fajardo et al., 2018). In this current study, we found lower PC:PE ratio for WB compared to N, which coincided with the upregulation of SERCA proteins in WB samples as shown in our proteomic data. Additionally, Patel and Witt (2017) showed that PE species with polyunsaturated acyl chains, such as 20:4 and 22:4, in the ER membrane can trigger the formation of hydroperoxides and induce cell apoptosis. Hydroperoxides in lipids can be deleterious to cells because they can disrupt the structure and function of the membrane (Girotti, 1998). Chao et al. (2020)’s product ion analysis result showed that the major fatty acid for the altered PE species 36:2 is linoleic acid, which is an omega 6 fatty acid and is characterized by its pro-inflammatory response. Increased inflammation has been observed in WB birds (Young and Rasmussen, 2020; Xing et al., 2021), and it is possible the excess omega 6 fatty acids in WB SR membrane indicated the WB broilers could be suffering from distress.

The LPC is derived from the cleaving of PC from PLA2 activity (Law et al., 2019). Our findings on LPC are supported by the previously discussed proteomics data on the upregulation of PLA2 in WB samples. Furthermore, PLA2 activity is calcium-dependent (Murakami and Kudo, 2002), and WB has been consistently shown to have elevated levels of calcium in our and many other studies (Soglia et al., 2016, 2018; Tasoniero et al., 2016). Therefore, we hypothesize that the high calcium level in WB PM may further contribute to the increase in PLA2 activity resulting in SR membrane phospholipid hydrolysis. This membrane integrity damage could play a role in the increased calcium release from the SR and potentially affect the function of the proteins embedded in the SR membrane (Cournia et al., 2015).

## Conclusion

This research begins to fill in the blanks of many texture defects observed in WB PM, particularly around collagen characteristics. Understanding the composition of mature collagen crosslinks in WB may partly explain the tough/rubbery texture of WB PM. The elevated levels of calcium found in WB PM could also play a role in the mechanism behind WB textural abnormalities. Our data indicated WB SR proteins are likely functioning as intended to mitigate the noted calcium imbalance. However, there are noted alterations to the phospholipid membrane composition and increased lipid catabolism in the WB SR, which could be contributing to increased calcium release due to a compromised structure. Finally, the significant upregulation of cholinesterase suggested there may be a source of cholinesterase inhibitors preventing the breakdown of acetylcholine and delaying the termination of action potential. If action potential is prolonged, more calcium would be expected to be released from the SR and could aid in explaining the high calcium levels observed in WB. While the current study provided novel data in the textural defects and the functionality/integrity of the SR of WB PM, many unknowns remain, which warrant further investigation to shed light on the etiology of WB myopathy.

## Data Availability Statement

The raw data supporting the conclusions of this article will be made available by the authors, without undue reservation. The proteomics data presented in the study are deposited in the ProteomeXchange repository, accession number PXD031445.

## Author Contributions

AW: data curation and writing—original draft preparation. WW, RM, and DB: data curation. MC: conceptualization, methodology, formal analysis, writing—original draft, and writing—review and editing. TO and EG: methodology and writing—review and editing. SH: data curation and methodology. BB: conceptualization and writing—review and editing. HZ: writing—review and editing. All authors contributed to the article and approved the submitted version.

## Conflict of Interest

The authors declare that the research was conducted in the absence of any commercial or financial relationships that could be construed as a potential conflict of interest.

## Publisher’s Note

All claims expressed in this article are solely those of the authors and do not necessarily represent those of their affiliated organizations, or those of the publisher, the editors and the reviewers. Any product that may be evaluated in this article, or claim that may be made by its manufacturer, is not guaranteed or endorsed by the publisher.

## Footnotes

^1^ https://david.ncifcrf.gov/

## References

[ref1] Abasht B. Mutryn M. F. Michalek R. D. Lee W. R. (2016). Oxidative stress and metabolic perturbations in wooden breast disorder in chickens. PLoS One 11:e0153750. doi: 10.1371/journal.pone.0153750, PMID: 27097013 PMC4838225

[ref2] Alvarez-Curto E. Inoue A. Jenkins L. Raihan S. Z. Prihandoko R. Tobin A. B.. (2016). Targeted elimination of G proteins and arrestins defines their specific contributions to both intensity and duration of G protein-coupled receptor signaling. J. Biol. Chem. 291, 27147–27159. doi: 10.1074/jbc.M116.754887, PMID: 27852822 PMC5207144

[ref3] Babu G. J. Wheeler D. Alzate O. Periasamy M. (2004). Solubilization of membrane proteins for two-dimensional gel electrophoresis: identification of sarcoplasmic reticulum membrane proteins. Anal. Biochem. 325, 121–125. doi: 10.1016/j.ab.2003.10.024, PMID: 14715292

[ref500] Baldi G. F. Soglia L. Laghi S. Tappi P. Rocculi S. Tavaniello D.. (2019). Comparison of quality traits among breast meat affected by current muscle abnormalities. Int. Food. Res. J. 115, 369–376. doi: 10.1016/j.foodres.2018.11.020, PMID: 30599954

[ref4] Baldi G. Yen C.-N. Daughtry M. R. Bodmer J. Bowker B. Zhuang H.. (2020). Exploring the factors contributing to the high ultimate pH of broiler Pectoralis major muscles affected by wooden breast condition. Front. Physiol. 11:343. doi: 10.3389/fphys.2020.00343, PMID: 32457639 PMC7227419

[ref5] Barone V. Randazzo D. Del Re V. Sorrentino V. Rossi D. (2015). Organization of junctional sarcoplasmic reticulum proteins in skeletal muscle fibers. J. Muscle Res. Cell Motil. 36, 501–515. doi: 10.1007/s10974-015-9421-5, PMID: 26374336

[ref6] Bellinger A. M. Mongillo M. Marks A. R. (2008). Stressed out: the skeletal muscle ryanodine receptor as a target of stress. J. Clin. Investig. 118, 445–453. doi: 10.1172/JCI34006, PMID: 18246195 PMC2214709

[ref7] Biswas A. K. Tandon S. (2019). “Casein zymography for analysis of calpain-1 and calpain-2 activity,” in Calpain Methods Molecular Biology, Vol. 1915. ed. Messer J. (New York, NY: Springer), 31–38. 10.1007/978-1-4939-8988-1_3 30617793

[ref8] Bowker B. Zhuang H. Barton E. Sanchez J. A.-M. (2016). “White striping and wooden breast defects influence meat quality and muscle protein characteristic in broiler breast meat.” in *Proceedings of 62nd International Congress of Meat Science and Technology*, Bangkok, Thailand.

[ref9] Boye T. L. Nylandsted J. (2016). Annexins in plasma membrane repair. Biol. Chem. 397, 961–969. doi: 10.1016/bs.ctm.2019.07.006 27341560

[ref10] Brasselet C. Durand E. Addad F. Zen A. A. H. Smeets M. B. Laurent-Maquin D.. (2005). Collagen and elastin cross-linking: a mechanism of constrictive remodeling after arterial injury. Am. J. Physiol. Heart Circ. Physiol. 289, H2228–H2233. doi: 10.1152/ajpheart.00410.2005, PMID: 15951346

[ref11] Burke J. E. Dennis E. A. (2009). Phospholipase A2 structure/function, mechanism, and signaling. J. Lipid Res. 50, S237–S242. doi: 10.1194/jlr.R800033-JLR200, PMID: 19011112 PMC2674709

[ref12] Bursztajn S. Schneider L. W. Jong Y.-J. Berman S. A. (1991). Calcium and ionophore A23187 stimulates deposition of extracellular matrix and acetylcholinesterase release in cultured myotubes. Cell Tissue Res. 265, 95–103. doi: 10.1007/BF00318143, PMID: 1913783

[ref13] Byron M. Von Staden M. E. Zhang X. Crist C. A. Zhai W. Schilling M. W. (2019). Impact of refrigerated storage on the dissipation of woody broiler breast meat. Meat Muscle Biol. 3, 74–74. doi: 10.22175/mmb2019.0074

[ref14] Cai K. Shao W. Chen X. Campbell Y. Nair M. Suman S.. (2018). Meat quality traits and proteome profile of woody broiler breast (pectoralis major) meat. Poult. Sci. 97, 337–346. doi: 10.3382/ps/pex284, PMID: 29053841

[ref15] Chao M. D. Domenech-Pérez K. Calkins C. R. (2017). Feeding vitamin E may reverse sarcoplasmic reticulum membrane instability caused by feeding wet distillers grains plus solubles to cattle. Prof. Anim. Sci. 33, 12–23. doi: 10.15232/pas.2016-01569

[ref16] Chao M. D. Donaldson E. A. Wu W. Welter A. A. O’Quinn T. G. Hsu W.-W.. (2020). Characterizing membrane phospholipid hydrolysis of pork loins throughout three aging periods. Meat Sci. 163:108065. doi: 10.1016/j.meatsci.2020.108065, PMID: 31986363 PMC7958495

[ref17] Chelu M. G. Danila C. I. Gilman C. P. Hamilton S. L. (2004). Regulation of ryanodine receptors by FK506 binding proteins. Trends Cardiovasc. Med. 14, 227–234. doi: 10.1016/j.tcm.2004.06.003 15451514

[ref18] Chun C. Welti R. Roth M. R. Richards M. Chao M. (2021). “Exploring the potential effect of anti-phospholipase a2 antibody to extend beef shelf-life in a beef liposome model system.” in *74th Recipient Meat Conference Proceedings Reno*, Nevada. 10.1016/j.meatsci.2022.109091 36587462

[ref19] Chun C. Wu W. Welter A. O’Quinn T. Magnin-Bissel G. Boyle D.. (2020). A preliminary investigation of the contribution of different tenderness factors to beef loin, tri-tip and heel tenderness. Meat Sci. 170:108247. doi: 10.1016/j.meatsci.2020.108247, PMID: 32736289

[ref20] Cipta S. Patel H. H. (2009). Molecular bandages: inside-out, outside-in repair of cellular membranes. Focus on “myoferlin is critical for endocytosis in endothelial cells.” Am. Physiol. Soc. 297, C481–C483. doi: 10.1152/ajpcell.00288.2009, PMID: 19587215 PMC2740387

[ref21] Colovic M. B. Krstic D. Z. Lazarevic-Pasti T. D. Bondzic A. M. Vasic V. M. (2013). Acetylcholinesterase inhibitors: pharmacology and toxicology. Curr. Neuropharmacol. 11, 315–335. doi: 10.2174/1570159X11311030006, PMID: 24179466 PMC3648782

[ref22] Cournia Z. Allen T. W. Andricioaei I. Antonny B. Baum D. Brannigan G.. (2015). Membrane protein structure, function, and dynamics: a perspective from experiments and theory. J. Membr. Biol. 248, 611–640. doi: 10.1007/s00232-015-9802-0, PMID: 26063070 PMC4515176

[ref23] da Silva T. F. Sousa V. F. Malheiro A. R. Brites P. (2012). The importance of ether-phospholipids: a view from the perspective of mouse models. Biochim. Biophys. Acta 1822, 1501–1508. doi: 10.1016/j.bbadis.2012.05.014, PMID: 22659211

[ref24] Diaz B. L. Arm J. P. (2003). Phospholipase A2. Prostaglandins, Leukot. Essent. Fat. Acids 69, 87–97. doi: 10.1016/S0952-3278(03)00069-3 12895591

[ref25] Doran P. Dowling P. Lohan J. McDonnell K. Poetsch S. Ohlendieck K. (2004). Subproteomics analysis of Ca^2+^−binding proteins demonstrates decreased calsequestrin expression in dystrophic mouse skeletal muscle. Eur. J. Biochem. 271, 3943–3952. doi: 10.1111/j.1432-1033.2004.04332.x, PMID: 15373840

[ref26] Dorninger F. Forss-Petter S. Wimmer I. Berger J. (2020). Plasmalogens, platelet-activating factor and beyond–ether lipids in signaling and neurodegeneration. Neurobiol. Dis. 145:10506. doi: 10.1016/j.nbd.2020.105061, PMID: 32861763 PMC7116601

[ref27] Dridi S. Kidd M. (2016). “Chapter 8: molecular pathways involved in amino acid and phosphorus utilization,” in Phytate Destruction-Consequences for Precision ANIMAL nutrition. eds. C. L. Walk, I. Kühn, H. H. Stein, M. T. Kidd, and M. Rodehutscord (Netherlands: Wageningen Academic), 119–128.

[ref28] Ertbjerg P. Puolanne E. (2017). Muscle structure, sarcomere length and influences on meat quality: a review. Meat Sci. 132, 139–152. doi: 10.1016/j.meatsci.2017.04.261, PMID: 28552497

[ref29] Fajardo V. A. Mikhaeil J. S. Leveille C. F. Tupling A. R. LeBlanc P. J. (2018). Elevated whole muscle phosphatidylcholine: phosphatidylethanolamine ratio coincides with reduced SERCA activity in murine overloaded plantaris muscles. Lipids Health Dis. 17, 47–48. doi: 10.1186/s12944-018-0687-7, PMID: 29534725 PMC5851149

[ref30] Farine L. Bütikofer P. (2013). The ins and outs of phosphatidylethanolamine synthesis in Trypanosoma brucei. Biochim. Biophys. Acta 1831, 533–542. doi: 10.1016/j.bbalip.2012.09.008, PMID: 23010476

[ref31] Fulton M. H. Key P. B. (2001). Acetylcholinesterase inhibition in estuarine fish and invertebrates as an indicator of organophosphorus insecticide exposure and effects. Environ. Toxicol. Chem. 20, 37–45. doi: 10.1002/etc.5620200104, PMID: 11351414

[ref32] Gilbert H. F. (1997). Protein disulfide isomerase and assisted protein folding. J. Biol. Chem. 272, 29399–29402. doi: 10.1074/jbc.272.47.29399 9367991

[ref33] Girotti A. W. (1998). Lipid hydroperoxide generation, turnover, and effector action in biological systems. J. Lipid Res. 39, 1529–1542. doi: 10.1016/S0022-2275(20)32182-9, PMID: 9717713

[ref34] Greene E. Cauble R. Dhamad A. E. Kidd M. T. Kong B. Howard S. M.. (2020). Muscle metabolome profiles in woody breast-(un) affected broilers: effects of quantum blue phytase-enriched diet. Front. Vet. Sci. 7:458. doi: 10.3389/fvets.2020.00458, PMID: 32851035 PMC7417653

[ref35] Hasegawa Y. Hara T. Kawasaki T. Yamada M. Watanabe T. Iwasaki T. (2020). Effect of wooden breast on postmortem changes in chicken meat. Food Chem. 315:126285. doi: 10.1016/j.foodchem.2020.126285, PMID: 32007816

[ref36] Horgan D. J. King N. L. Kurth L. B. Kuypers R. (1990). Collagen crosslinks and their relationship to the thermal properties of calf tendons. Arch. Biochem. Biophys. 281, 21–26. doi: 10.1016/0003-9861(90)90407-p, PMID: 2383022

[ref37] Inagi R. (2010). Endoplasmic reticulum stress as a progression factor for kidney injury. Curr. Opin. Pharmocol. 10, 156–165. doi: 10.1016/j.coph.2009.11.006 20045381

[ref38] Kanno K. Wu M. K. Scapa E. F. Roderick S. L. Cohen D. E. (2007). Structure and function of phosphatidylcholine transfer protein (PC-TP)/StarD2. Biochim. Biophys. Acta 1771, 654–662. doi: 10.1016/j.bbalip.2007.04.003, PMID: 17499021 PMC2743068

[ref39] Kim K. M. Kirn D. K. Park Y. M. Kim C.-K. Na D. S. (1994). Annexin-I inhibits phospholipase A2 by specific interaction, not by substrate depletion. FEBS Lett. 343, 251–255. doi: 10.1016/0014-5793(94)80566-0, PMID: 8174710

[ref40] Koohmaraie M. Geesink G. (2006). Contribution of postmortem muscle biochemistry to the delivery of consistent meat quality with particular focus on the calpain system. Meat Sci. 74, 34–43. doi: 10.1016/j.meatsci.2006.04.025, PMID: 22062714

[ref41] Kurebayashi N. Takeshima H. Nishi M. Murayama T. Suzuki E. Ogawa Y. (2003). Changes in Ca^2+^ handling in adult MG29-deficient skeletal muscle. Biochem. Biophys. Res. Commun. 310, 1266–1272. doi: 10.1016/j.bbrc.2003.09.146, PMID: 14559251

[ref42] Kuttappan V. A. Bottje W. Ramanathan R. Hartson S. D. Coon C. N. Kong B.-W.. (2017). Proteomic analysis reveals changes in carbohydrate and protein metabolism associated with broiler breast myopathy. Poult. Sci. 96, 2992–2999. doi: 10.3382/ps/pex069, PMID: 28499042

[ref43] Kuttappan V. Hargis B. Owens C. (2016). White striping and woody breast myopathies in the modern poultry industry: a review. Poult. Sci. 95, 2724–2733. doi: 10.3382/ps/pew216, PMID: 27450434

[ref44] Lambert I. Pedersen S. Poulsen K. (2006). Activation of PLA2 isoforms by cell swelling and ischaemia/hypoxia. Acta Physiol. 187, 75–85. doi: 10.1111/j.1748-1716.2006.01557.x, PMID: 16734744

[ref45] Lanner J. T. Georgiou D. K. Joshi A. D. Hamilton S. L. (2010). Ryanodine receptors: structure, expression, molecular details, and function in calcium release. Cold Spring Harb. Perspect. Biol. 2:a003996. doi: 10.1101/cshperspect.a003996, PMID: 20961976 PMC2964179

[ref46] Law S.-H. Chan M.-L. Marathe G. K. Parveen F. Chen C.-H. Ke L.-Y. (2019). An updated review of lysophosphatidylcholine metabolism in human diseases. Int. J. Mol. Sci. 20:1149. doi: 10.3390/ijms20051149, PMID: 30845751 PMC6429061

[ref47] Lee H. Sante-Lhoutellier V. Vigouroux S. Briand Y. Briand M. (2008). Role of calpains in postmortem proteolysis in chicken muscle. Poult. Sci. 87, 2126–2132. doi: 10.3382/ps.2007-00296, PMID: 18809876

[ref48] Liu Z. Du X. Yin C. Chang Z. (2013). Shotgun proteomic analysis of sarcoplasmic reticulum preparations from rabbit skeletal muscle. Proteomics 13, 2335–2338. doi: 10.1002/pmic.201200138, PMID: 23713034

[ref49] Liu J. Puolanne E. Schwartzkopf M. Arner A. (2020). Altered sarcomeric structure and mechanics in woody breast myopathy in avian pectoralis major. Front. Physiol. 11:287. doi: 10.3389/fphys.2020.00287, PMID: 32328000 PMC7160512

[ref50] Maddock K. Huff-Lonergan E. Rowe L. Lonergan S. M. (2005). Effect of pH and ionic strength on μ-and m-calpain inhibition by calpastatin. J. Anim. Sci. 83, 1370–1376. doi: 10.2527/2005.8361370x, PMID: 15890814

[ref51] Marchesi J. A. P. Ibelli A. M. G. Peixoto J. O. Cantão M. E. Pandolfi J. R. C. Marciano C. M. M.. (2019). Whole transcriptome analysis of the pectoralis major muscle reveals molecular mechanisms involved with white striping in broiler chickens. Poult. Sci. 98, 590–601. doi: 10.3382/ps/pey429, PMID: 30239913

[ref52] McGleenon B. Dynan K. Passmore A. (1999). Acetylcholinesterase inhibitors in Alzheimer’s disease. Br. J. Clin. Pharmacol. 48:471, –480. doi: 10.1046/j.1365-2125.1999.00026.x, PMID: 10583015 PMC2014378

[ref53] Morel J. L. Rakotoarisoa L. Jeyakumar L. H. Fleischer S. Mironneau C. Mironneau J. (2004). Decreased expression of ryanodine receptors alters calcium-induced calcium release mechanism in mdx duodenal myocytes. J. Biol. Chem. 279, 21287–21293. doi: 10.1074/jbc.M311124200, PMID: 14985349

[ref54] Mouchlis V. D. Chen Y. McCammon J. A. Dennis E. A. (2018). Membrane allostery and unique hydrophobic sites promote enzyme substrate specificity. J. Am. Chem. Soc. 140, 3285–3291. doi: 10.1021/jacs.7b12045, PMID: 29342349 PMC5846079

[ref55] Mudalal S. Lorenzi M. Soglia F. Cavani C. Petracci M. (2015). Implications of white striping and wooden breast abnormalities on quality traits of raw and marinated chicken meat. Animal. 9, 728–734. doi: 10.1017/S175173111400295X, PMID: 25500004

[ref56] Murakami M. Kudo I. (2002). Phospholipase A2. J. Biochem. 131, 285–292. doi: 10.1093/oxfordjournals.jbchem.a003101 11872155

[ref57] Mutryn M. F. Brannick E. M. Fu W. Lee W. R. Abasht B. (2015). Characterization of a novel chicken muscle disorder through differential gene expression and pathway analysis using RNA-sequencing. BMC Genomics 16:399. doi: 10.1186/s12864-015-1623-0, PMID: 25994290 PMC4438523

[ref58] Ohlendieck K. (2013). The pathophysiological role of impaired calcium handling in muscular dystrophy- Madame Curie Bioscience Database. Landes Bioscience, TX. Available from: https://www.ncbi.nlm.nih.gov/books/NBK6173/ (Accessed June 10, 2021).

[ref59] Papah M. B. Brannick E. M. Schmidt C. J. Abasht B. (2018). Gene expression profiling of the early pathogenesis of wooden breast disease in commercial broiler chickens using RNA-sequencing. PLoS One 13:e0207346. doi: 10.1371/journal.pone.0207346, PMID: 30517117 PMC6281187

[ref60] Patel D. Witt S. N. (2017). Ethanolamine and phosphatidylethanolamine: partners in health and disease. Oxid. Med. Cell Longev. 2017:4829180. doi: 10.1155/2017/4829180, PMID: 28785375 PMC5529665

[ref61] Periasamy M. Kalyanasundaram A. (2007). SERCA pump isoforms: their role in calcium transport and disease. Muscle Nerve 35, 430–442. doi: 10.1002/mus.20745, PMID: 17286271

[ref62] Petracci M. Mudalal S. Soglia F. Cavani C. (2015). Meat quality in fast-growing broiler chickens. Worlds Poult. Sci. J. 71, 363–374. doi: 10.1017/S0043933915000367

[ref63] Puolanne T. E. J. Costandache C. G. Ertbjerg P. (2021). Influence of woody breast myopathy on sarcomere length and tensile strength in commercial broiler pectoralis major muscle. Meat Muscle Biol. 5, 1–11. doi: 10.22175/mmb.11564

[ref64] Putney J. W. Tomita T. (2012). Phospholipase C signaling and calcium influx. Adv. Biol. Regul. 52, 152–164. doi: 10.1016/j.advenzreg.2011.09.005, PMID: 21933679 PMC3560308

[ref65] Quinn D. M. (1987). Acetylcholinesterase: enzyme structure, reaction dynamics, and virtual transition states. Chem. Rev. 87, 955–979. doi: 10.1021/cr00081a005

[ref66] Rao R. V. Bredesen D. E. (2004). Misfolded proteins, endoplasmic reticulum stress and neurodegeneration. Curre. Opin. Cell Biol. 16, 653–662. doi: 10.1016/j.ceb.2004.09.012, PMID: 15530777 PMC3970707

[ref67] Rossi D. Barone V. Giacomello E. Cusimano V. Sorrentino V. (2008). The sarcoplasmic reticulum: an organized patchwork of specialized domains. Traffic 9, 1044–1049. doi: 10.1111/j.1600-0854.2008.00717.x, PMID: 18266914

[ref68] Roth D. Lynes E. Riemer J. Hansen H. G. Althaus N. Simmen T.. (2010). A di-arginine motif contributes to the ER localization of the type I transmembrane ER oxidoreductase TMX4. Biochem. J. 425, 195–208. doi: 10.1042/BJ20091064 19811453

[ref69] Ruberg M. Rieger F. Villageois A. Bonnet A. M. Agid Y. (1986). Acetylcholinesterase and butyrylcholinesterase in frontal cortex and cerebrospinal fluid of demented and non-demented patients with Parkinson’s disease. Brain Res. 362, 83–91. doi: 10.1016/0006-8993(86)91401-0, PMID: 3942870

[ref70] Rubio J. M. Rodríguez J. P. Gil-de-Gómez L. Guijas C. Balboa M. A. Balsinde J. (2015). Group V secreted phospholipase A2 is upregulated by IL-4 in human macrophages and mediates phagocytosis via hydrolysis of ethanolamine phospholipids. J. Immunol. 194, 3327–3339. doi: 10.4049/jimmunol.1401026, PMID: 25725101

[ref71] Sberna G. Sáez-Valero J. Beyreuther K. Masters C. L. Small D. H. (1997). The amyloid β-protein of Alzheimer’s disease increases acetylcholinesterase expression by increasing intracellular calcium in embryonal carcinoma P19 cells. J. Neurosci. 69, 1177–1184. doi: 10.1046/j.1471-4159.1997.69031177.x, PMID: 9282941

[ref72] Schreurs F. (2000). Post-mortem changes in chicken muscle. Worlds Poult. Sci. J. 56, 319–346. doi: 10.1079/WPS20000023

[ref73] Sihvo H.-K. Immonen K. Puolanne E. (2014). Myodegeneration with fibrosis and regeneration in the pectoralis major muscle of broilers. Vet. Pathol. 51, 619–623. doi: 10.1177/0300985813497488, PMID: 23892375

[ref74] Soglia F. Mudalal S. Babini E. Di Nunzio M. Mazzoni M. Sirri F.. (2016). Histology, composition, and quality traits of chicken Pectoralis major muscle affected by wooden breast abnormality. Poult. Sci. 95, 651–659. doi: 10.3382/ps/pev353, PMID: 26706363

[ref75] Soglia F. Zeng Z. Gao J. Puolanne E. Cavani C. Petracci M.. (2018). Evolution of proteolytic indicators during storage of broiler wooden breast meat. Poult. Sci. 97, 1448–1455. doi: 10.3382/ps/pex398, PMID: 29300955

[ref76] Solo J. (2016). Meat quality and sensory analysis of broiler breast fillets with woody breast muscle myopathy. Graduate Theses and Dissertations Retrieved from Available at: https://scholarworks.uark.edu/etd/1603

[ref77] Sun X. Koltes D. Coon C. Chen K. Owens C. (2018). Instrumental compression force and meat attribute changes in woody broiler breast fillets during short-term storage. Poult. Sci. 97, 2600–2606. doi: 10.3382/ps/pey107, PMID: 29688538

[ref78] Tajhya R. B. Patel R. S. Beeton C. (2017). “Detection of matrix metalloproteinases by zymography,” in Matrix Metalloproteases Methods Molecular Biology. Vol. 1579. ed. Galea C. (New York, NY: Humana Press), 231–244. 10.1007/978-1-4939-6863-3_12 PMC5465868 28299740

[ref79] Tasoniero G. Bowker B. Zhuang H. (2020). Research note: texture characteristics of wooden breast fillets deboned at different postmortem times. Poult. Sci. 99, 4096–4099. doi: 10.1016/j.psj.2020.04.028, PMID: 32731997 PMC7597905

[ref80] Tasoniero G. Cullere M. Cecchinato M. Puolanne E. Dalle Zotte A. (2016). Technological quality, mineral profile, and sensory attributes of broiler chicken breasts affected by white striping and wooden breast myopathies. Poult. Sci. 95, 2707–2714. doi: 10.3382/ps/pew215, PMID: 27486252

[ref81] Tijare V. V. Yang F. Kuttappan V. Alvarado C. Coon C. Owens C. (2016). Meat quality of broiler breast fillets with white striping and woody breast muscle myopathies. Poult. Sci. 95, 2167–2173. doi: 10.3382/ps/pew129, PMID: 27081195

[ref82] Tornberg E. (2005). Effects of heat on meat proteins–implications on structure and quality of meat products. Meat Sci. 70, 493–508. doi: 10.1016/j.meatsci.2004.11.021, PMID: 22063748

[ref83] Torrescano G. Sanchez-Escalante A. Gimenez B. Roncales P. Beltrán J. A. (2003). Shear values of raw samples of 14 bovine muscles and their relation to muscle collagen characteristics. Meat Sci. 64, 85–91. doi: 10.1016/S0309-1740(02)00165-1, PMID: 22062666

[ref84] van der Veen J. N. Kennelly J. P. Wan S. Vance J. E. Vance D. E. Jacobs R. L. (2017). The critical role of phosphatidylcholine and phosphatidylethanolamine metabolism in health and disease. Biochim. Biophys. Acta 1859, 1558–1572. doi: 10.1016/j.bbamem.2017.04.006, PMID: 28411170

[ref85] Vance J. E. (2015). Phospholipid synthesis and transport in mammalian cells. Traffic 16, 1–18. doi: 10.1111/tra.12230 25243850

[ref86] Vierck K. R. O’Quinn T. G. Noel J. A. Houser T. A. Boyle E. A. Gonzalez J. M. (2018). Effects of marbling texture on muscle fiber and collagen characteristics. Meat Muscle Biol. 2, 75–82. doi: 10.22175/mmb2017.10.0054

[ref87] Villa A. Podini P. Nori A. Panzeri M. C. Martini A. Meldolesi J.. (1993). The endoplasmic reticulum-sarcoplasmic reticulum connection. Exp. Cell Res. 209, 140–148. doi: 10.1006/excr.1993.1294, PMID: 8223998

[ref88] Visse R. Nagase H. (2003). Matrix metalloproteinases and tissue inhibitors of metalloproteinases: structure, function, and biochemistry. Circ. Res. 92, 827–839. doi: 10.1161/01.RES.0000070112.80711.3D 12730128

[ref89] Vu H. S. Roston R. Shiva S. Hur M. Wurtele E. S. Wang X.. (2015). Modifications of membrane lipids in response to wounding of Arabidopsis thaliana leaves. Plant Signal. Behav. 10:e1056422. doi: 10.1080/15592324.2015.1056422, PMID: 26252884 PMC4883853

[ref90] Wagner M. Rudakova E. Schütz V. Frank M. Ehmke H. Volk T. (2010). Larger transient outward K+ current and shorter action potential duration in Gα 11 mutant mice. Pflügers Arch.-Eur. J. Physiol. 459, 607–618. doi: 10.1007/s00424-009-0762-z 19953263

[ref91] Wilkie G. S. Schirmer E. C. (2008). Purification of nuclei and preparation of nuclear envelopes from skeletal muscle. Methods Mol. Bio. 463, 23–41. doi: 10.1007/978-1-59745-406-3_2, PMID: 18951158

[ref92] Wu W. J. Welter A. A. Rice E. A. Olson B. A. O’Quinn T. G. Boyle E. A.. (2021). Biochemical factors affecting east Asian consumers’ sensory preferences of six beef shank cuts. Meat Muscle Biol. 5, 1–18. doi: 10.22175/mmb.11626

[ref93] Xiao S. Gao W. Chen Q.-F. Chan S.-W. Zheng S.-X. Ma J.. (2010). Overexpression of Arabidopsis acyl-CoA binding protein ACBP3 promotes starvation-induced and age-dependent leaf senescence. Plant Cell 22, 1463–1482. doi: 10.1105/tpc.110.075333, PMID: 20442372 PMC2899868

[ref94] Xing T. Pan X. Zhang L. Gao F. (2021). Hepatic oxidative stress, apoptosis and inflammation in broiler chickens with wooden breast myopathy. Front. Physiol. 12:415. doi: 10.3389/fphys.2021.659777, PMID: 33935806 PMC8081064

[ref95] Yalcin S. Ozkan S. Acar M. C. Meral O. (2018). The occurrence of deep pectoral myopathy in broilers and associated changes in breast meat quality. British Poult. Sci. 59, 55–62. doi: 10.1080/00071668.2017.1401214, PMID: 29113457

[ref96] Yang F.-W. Wang H. Wang C. Chi G.-N. (2020). Upregulation of acetylcholinesterase caused by downregulation of microRNA-132 is responsible for the development of dementia after ischemic stroke. J. Cell. Biochem. 121, 135–141. doi: 10.1002/jcb.28985, PMID: 31578769

[ref97] Young J. F. Rasmussen M. K. (2020). Differentially expressed marker genes and glycogen levels in pectoralis major of Ross308 broilers with wooden breast syndrome indicates stress, inflammation and hypoxic conditions. Food Chem. Mol. Sci. 1:100001. doi: 10.1016/j.fochms.2020.100001 PMC8991981 35415620

[ref98] Zhang L. Kelley J. Schmeisser G. Kobayashi Y. M. Jones L. R. (1997). Complex formation between junctin, triadin, calsequestrin, and the ryanodine receptor: proteins of the cardiac junctional sarcoplasmic reticulum membrane. J. Biol. Chem. 272, 23389–23397. doi: 10.1074/jbc.272.37.23389 9287354

[ref99] Zhou Z. Marepally S. R. Nune D. S. Pallakollu P. Ragan G. Roth M. R.. (2011). LipidomeDB data calculation environment: online processing of direct-infusion mass spectral data for lipid profiles. Lipids 46, 879–884. doi: 10.1007/s11745-011-3575-8, PMID: 21647782 PMC3312010

